# Evaluation of osteoconductivity and inflammatory response of a resin-based and a resin-free calcium silicate sealer: an in vivo study

**DOI:** 10.1007/s00784-025-06417-y

**Published:** 2025-06-05

**Authors:** Mohamed Ahmed Elsayed, Abeer AbdElhakim Elgendy, Ihab Elsayed Hassanien, Edgar Schäfer

**Affiliations:** 1 Department of Endodontics, RAK College of Dental Sciences, RAK Medical and Health Sciences University, Ras Al-Khaimah, United Arab Emirates; 2 Department of Endodontics, Faculty of Dentistry, Assiut University, Assiut, Egypt; 3 Department of Endodontics, Faculty of Dentistry, Ain Shams University, Cairo, Egypt; 4 Department of Endodontics, Faculty of Dentistry, El Gallala University, Suez, Egypt; 5 Central Interdisciplinary Ambulance, School of Dentistry, University of Münster, Waldeyerstr. 30, D-48149 Münster, Germany

**Keywords:** Bioceramic sealer, Biocompatibility, Calcium silicate sealer, TotalFill BC, MTA fillapex, Periapical healing, Osteoconductivity, Inflammatory response, Immunohistochemistry

## Abstract

**Aim:**

This study aimed to assess the biocompatibility and osteoconductivity of a resin-based and a resin-free calcium silicate sealer and their ability to promote healing of induced periapical lesions in mature dogs’ teeth.

**Materials and methods:**

Periapical lesions were induced at 40 mandibular premolars of eight mongrel dogs. Following root canal preparation, the canals were obturated with a single cone and either TotalFill BC (*n* = 16) or MTA Fillapex (*n* = 16), while the positive control group (*n* = 8) remained unfilled. To ensure sealer extrusion during obturation, apical foramina were enlarged intentionally. Histological evaluations were conducted after one and four months, utilizing hematoxylin and eosin staining for inflammatory cell counts, Goldner’s trichrome stain for new bone formation, and immunohistochemistry for osteopontin (OPN) expression. ImageJ software was used for quantitative analysis, and statistical comparisons were made using the Kruskal-Wallis and the Mann-Whitney U with Bonferroni’s tests (*p* < 0.05).

**Results:**

TotalFill BC Sealer induced a significantly lower inflammatory response than MTA Fillapex (*p* < 0.05). A significant reduction in inflammation was observed over time. TotalFill BC Sealer exhibited a significantly higher percentage of new bone formation and significantly higher OPN expression than MTA Fillapex and the positive control (*p* < 0.05). At four months, TotalFill BC Sealer showed enhanced bone formation with minimal residual inflammation, whereas MTA Fillapex demonstrated moderate healing with ongoing remodeling.

**Conclusion:**

The resin-free TotalFill BC Sealer exhibited superior biocompatibility, promoted faster inflammation resolution, and stimulated more new bone formation compared to the resin-based sealer MTA Fillapex when in direct contact with periapical tissues.

## Introduction

Root canal sealers play a crucial role in achieving successful root canal treatment by ensuring a bacteria-tight seal and promoting periapical healing. Since sealers are designed for direct contact with the periapical tissues, there is always concern about potential adverse tissue reactions [1]. The ideal root canal sealer should possess excellent sealing ability, dimensional stability, insolubility in tissue fluids, adequate adhesion to canal walls, and biocompatibility. However, many conventional sealers have demonstrated varying degrees of cytotoxicity and solubility over time, leading to potential leakage and compromised long-term outcomes [2].

The introduction of bioactive materials, particularly mineral trioxide aggregate (MTA), revolutionized endodontic therapy due to their superior biocompatibility, osteogenic potential, and ability to promote periapical healing [3]. The capacity of MTA to induce favorable tissue responses and stimulate mineralized tissue repair has made MTA a preferred choice for perforation repair, root-end filling, and pulp capping [4]. However, its long setting time, potential for dentin discoloration, and difficult handling characteristics have limited its direct use as a root canal sealer [5]. To overcome these drawbacks, MTA-based sealers, such as MTA Fillapex (Angelus Solucões Odontológicas, Londrina, PR, Brazil), were developed by incorporating resins and thickening agents to improve flowability and handling. MTA Fillapex is a paste-catalyst sealer. Paste A is composed of salicylate resin, bismuth oxide, and fumed silica. Paste B includes fumed silicon dioxide, titanium dioxide, base resin, and MTA (Table 1). Although it contains MTA, concerns regarding their inflammatory response and slower tissue healing have been reported [1].

**Table 1:** Manufacturers, lot numbers, and composition of the tested sealers

Sealer	manufacturer (Lot number)		Composition (Approximate Percentage)
TotalFill BC	FKG Dentaire, La Chaux-de-Fonds, Switzerland (4003SP-V 09/2017).	Zirconium oxide (25–40%) Tricalcium silicate (25–35%) Dicalcium silicate (7–15%) Calcium Phosphate (5–10%) Calcium hydroxide (1 − 10%) Fillers (5–15%) Water-Free Vehicle (< 5%)	
MTA Fillapex	Angelus, Londrina, PR, Brazil (35867, 35760, 35267-V 05/2017)	**Paste A**	
		Salicylate resin (40–50%) Bismuth trioxide (5–10%) Nanoparticulated silica (20–25%) Pigments (5–10%)	
		**Paste B**	
		Base resin (5–10%) Fumed Silica (5–10%) Titanium Dioxide (< 5%) MTA (13–15%)	

Calcium silicate-based sealers, also known as bioceramic sealers, have gained increasing attention due to their bioactivity, excellent sealing properties, and enhanced biocompatibility [6]. TotalFill BC (FKG Dentaire, La Chaux-de-Fonds, Switzerland) is a premixed, nanoparticle-based, bioceramic paste that sets in the presence of moisture and releases calcium ions, which are critical for osteogenesis. TotalFill BC is composed of zirconium oxide, tricalcium silicate, dicalcium silicate, colloidal silica, calcium silicates, monobasic calcium phosphate, and calcium hydroxide (Table 1). It has a working time of approximately four hours at room temperature and a setting time of about three hours and requires moisture during the setting process [6].

Several studies have explored the interaction of endodontic sealers with periapical tissues, emphasizing the importance of their biocompatibility and osteogenic potential [7]. Inflammatory reactions induced by sealers can significantly affect periapical healing and bone regeneration. Excessive inflammatory responses may lead to delayed healing or periapical pathology, making it essential to assess the inflammatory potential of different sealers [2]. Osteogenesis, osteoinduction, and osteoconduction are various biological processes that enable the regeneration of lost bone. An osteoconductive material permits bone growth on its surface or down into pores or channels. Most osteoconductive materials are able to operate as a scaffold to guide osteoid tissue regeneration and release Ca^2+^ and other key ions for long durations [7]. Release of calcium ions enhances osteoblastic proliferation, differentiation, and extracellular matrix mineralization [8].

The evaluation of osteoconductivity and inflammatory response in a dynamic environment that mimics clinical conditions is essential to understanding the biological behavior of endodontic materials. While in vitro single-cell models are helpful for some controlled studies, they lack the dynamic and systemic influences present in a living organism. The in vivo models provide a more clinically relevant representation and account for comprehensive biological responses, as they mimic the complex interactions between different cell types, immune responses, and tissue healing processes [9]. They also consider the effects of mastication force and oral thermal fluctuations on material properties and tissue response.

Given the limited in vivo studies focusing on the biological properties of calcium silicate sealers, further evaluation of their osteoconductivity is warranted. For calcium silicate-based repair and pulp-capping materials, it has recently been reported that resin-free materials are more biocompatible and possess a greater potential to induce proliferation and adhesion of stem cells than resin-based calcium silicate-based materials [10, 11]. Similar studies comparing the effects of resin-based and resin-free calcium silicate-based sealers are scarce.

It was therefore the aim of this study to compare the biocompatibility and osteoconductivity of a resin-based (MTA Fillapex) and a resin-free (TotalFill BC Sealer) calcium silicate sealer and their ability to promote healing of induced periapical lesions in mature dogs’ teeth. The null hypothesis tested was that there would be no differences between the two sealers.

## Materials and methods

This study was approved by the Institutional Animal Care and Use Committee at Ain Shams University, Egypt (Approval No: FDASU-REC-151-2015) and adhered to ethical guidelines for the humane treatment of animals, including appropriate housing, medical care, and anesthesia protocols. The study objectives could not be achieved through non-animal models, and the minimum necessary number of animals was used to ensure statistical validity. The required sample size was determined using GPower software (Düsseldorf, Germany). A minimum of 75 samples was calculated to detect an effect size of 0.25 with a power of 85% (1-β = 0.85) and a partial eta-squared of 0.06 at a significance level of *p* < 0.05. To account for potential exclusions, 80 root samples from 40 mature mandibular premolars were included in the study.

### Animal selection and housing

The study was conducted on eight healthy adult mongrel dogs (aged 2–5 years), with an average weight of 14.5 ± 0.5 kg. Dogs of both sexes were used. Each animal underwent a thorough physical examination by a veterinarian to confirm health status and the absence of systemic disease. The animals were housed individually in well-ventilated, parasite-free galvanized steel cages in a quiet, controlled environment. They were fed a balanced diet of bread, meat, and chicken twice daily, with unrestricted access to water. A total of five mandibular premolars per dog were selected for the study: the right mandibular second, third, and fourth premolars, as well as the left third and fourth premolars. All experimental procedures were performed at the veterinary unit of Ain Shams University (Cairo, Egypt). Measures were taken to minimize pain, distress, and discomfort in accordance with established animal welfare guidelines.

### Induction of periapical pathosis

General anesthesia was administered after a 12-hour fasting period. Pre-medication included atropine sulfate (0.05 mg/kg; Atropine Sulfate^®^, ADWIA Co., Egypt) to reduce salivary secretions, along with intramuscular administration of acepromazine (0.04 mg/kg) and morphine sulfate (0.3 mg/kg) for sedation and analgesia. Anesthesia was induced with an injection of ketamine hydrochloride (10 mg/kg; Ketamine^®^ 5%, Sigma-Tec Co., Egypt) combined with xylazine hydrochloride (1 mg/kg). Maintenance anesthesia was achieved with incremental doses of thiopental sodium (25 mg/kg; Thiopental Sodium^®^, EIPICO, Egypt), and its depth was confirmed by the absence of the blink reflex. A canine mouth gag was used to maintain the mouth opening throughout the procedure.

Preoperative periapical radiographs were taken to confirm the apical closure of roots, assess periapical tissue health, and determine the provisional working length. Access cavities were prepared using a size 2 diamond bur (Dentsply Maillefer, Ballaigues, Switzerland) mounted on an electric micromotor handpiece (NSK, Nakanishi Inc., Tochigi, Japan). The pulp tissue was extirpated with barbed broaches and Hedstrom files (Mani Inc., Tochigi, Japan). To induce periapical periodontitis, root canals were left open to the oral cavity for seven days to allow bacterial contamination [12]. Additionally, a supragingival plaque from the dogs’ teeth was introduced into the pulp chambers to further ensure infection of the root canal system [13]. After this period, access cavities were sealed with glass ionomer filling (GC Fuji IX, GC America Inc., Alsip, IL, USA).

### Root canal instrumentation

After sixty days, periapical radiographs were taken to confirm the presence of periapical radiolucencies, indicating periapical lesion formation. During this period, dogs were fed a soft diet and received daily Carprofen (Rimadyl tab^®^, Pfizer Co., New York, NY, USA) at 4.4 mg/kg for analgesia. Under anesthesia and cotton roll isolation, previously infected teeth were re-accessed. The working length was initially determined using an apex locator (Ipex II, NSK, Tokyo, Japan), followed by verification with a radiograph. To standardize the apical foramen diameter and facilitate the formation of sealer puffs, the apical foramen was perforated and sequentially enlarged using K-files (Mani Inc., Tochigi, Japan) sizes 20, 25, and 30. Mechanical instrumentation of the root canals was performed up to the full working length using the ProTaper Universal system (Dentsply Maillefer, Ballaigues, Switzerland) up to #F5, followed by manual apical preparation with K-files (Mani Inc., Tochigi, Japan) up to size 70 to ensure the creation of an apical stop. After each file, canals were irrigated with 1 mL of 5.25% sodium hypochlorite using a 30-gauge needle (SuperEndo Disposable, Signel Biomedical Pvt., New Delhi, India) positioned 2 mm from the working length. A final irrigation with 5 mL of 5.25% sodium hypochlorite for 1 min was performed, followed by 1 mL of 17% EDTA (Prevest DenPro Limited, Jammu, India) for 1 min. Finally, canal dryness was achieved with paper points, and apical patency was maintained using a size 30 K file.

### Obturation

**Fig. 1:**
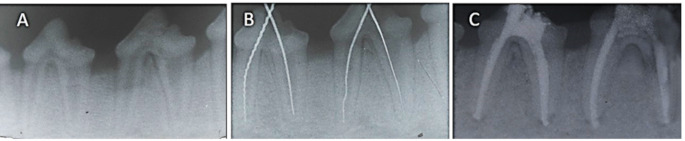
(**A**) Preoperative periapical radiographs were obtained to verify the apical closure of roots, evaluate the health of the periapical tissue, and establish the provisional working length. (**B**) Using radiography to determine and verify the working length. (**C**) Postoperative radiograph

The 40 teeth were divided into three groups: Group 1: Obturation with gutta-percha and TotalFill BC Sealer (*n* = 16). Group 2: Obturation with gutta-percha and MTA Fillapex (*n* = 16). Group 3 (Positive Control): Canals prepared but left un-obturated (*N* = 8). Each dog contributed to all experimental and control groups. Groups 1 and 2 were further divided based on observation periods: Subgroup A: 1-month evaluation (*n* = 8). Subgroup B: 4-month evaluation (*n* = 8). The control group was similarly subdivided into Subgroup A (*n* = 4) and Subgroup B (*n* = 4). Root canals were filled using a single-cone technique with size 70, 0.02 taper gutta-percha, and the assigned sealer. Sealers were applied via an intracanal tip, and postoperative radiographs confirmed a sealer extrusion of at least 1 mm beyond the apex to ensure contact with periapical tissues. Coronal access was restored with glass ionomer cement (GC Fuji IX) (Fig. 1).

### Tissue preparation and histologic analysis

**Fig. 2:**
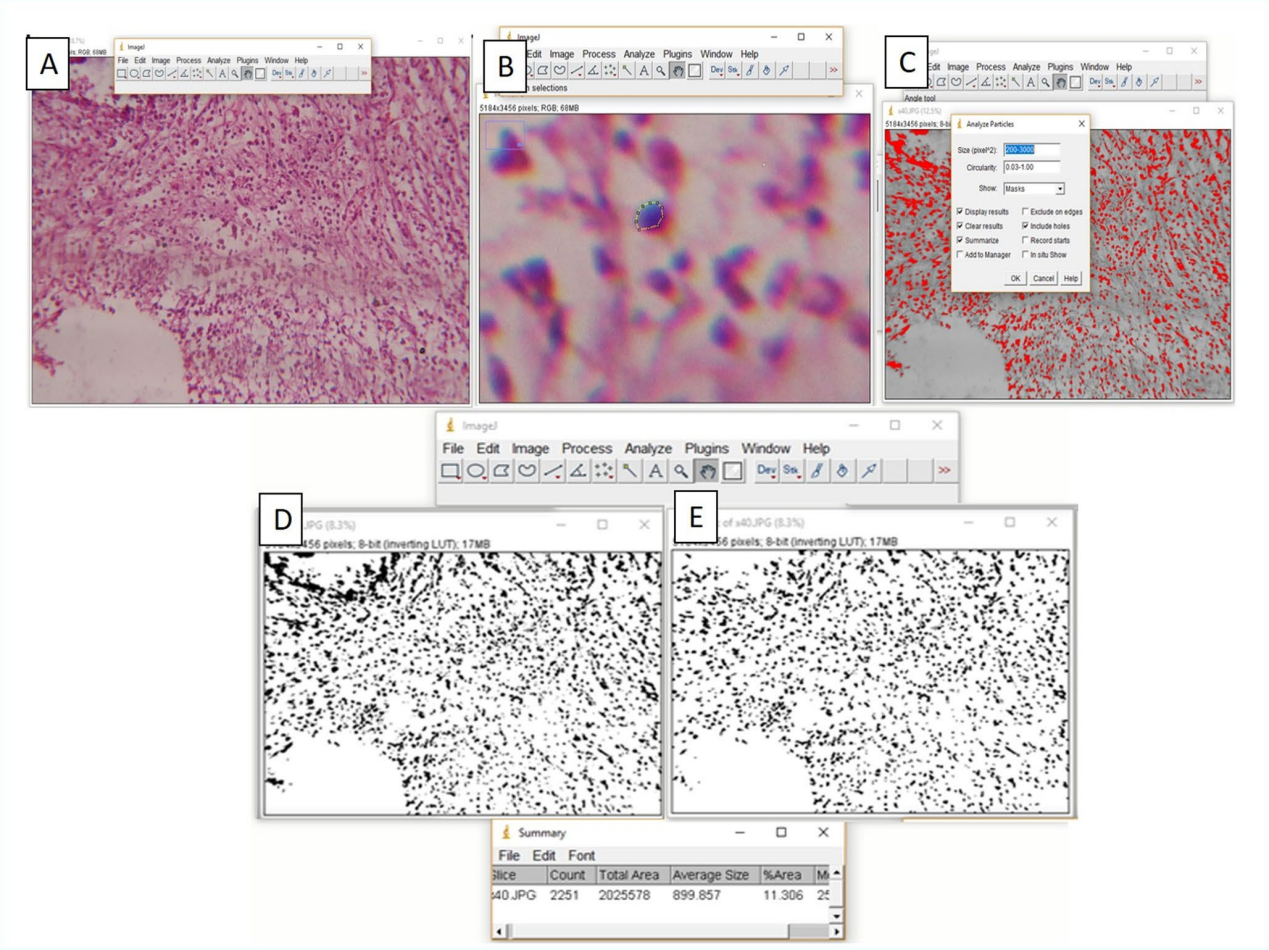
Digital inflammatory cell count. (**A**) ImageJ was used for image analysis. (**B**) The *polygon selection* tool was used to choose an inflammatory cell, and the average cell size was calculated by repeating the measuring procedure for five different-sized cells. (**C**) The circularity value and particle size range were defined using the *Analyze Particles* function in order to exclude undesirable structures. (**D**) The image was transformed to a binary (black-and-white) format using the *Threshold/Apply* function. (**E**) Following optimization, inflammatory cells were counted using the *Show Outlines* and *Summarize* functions, guaranteeing increased accuracy

Postoperatively, dogs were maintained on a soft diet (bread, broth, and chicken gut) and received 3 mL of diclofenac sodium for analgesia. At each time point (1 and 4 months), four randomly selected animals were euthanized via an intravenous overdose of thiopental sodium (30 mg/kg). Mandibles were dissected and sectioned using a precision saw (Isomet 4000, Bühler, Leinfelden-Echterdingen, Germany) and fixed in 10% neutral-buffered formalin for five days. Samples were decalcified in disodium EDTA (ADWIC, El Nasr Pharmaceutical Co., Cairo, Egypt) for three months before paraffin embedding. Three serial mesiodistal Sects. (3–5 μm thick) were prepared per root and surrounding bone tissue using a microtome (Leica RM 2165, Leica, Wetzlar, Germany) and mounted on glass slides. The sections were assigned to hematoxylin and eosin staining, Goldner’s Trichrome staining, and immunohistochemistry for analysis. A total of 240 histological sections were examined under a microscope (BX60, Olympus, Tokyo, Japan) by a blinded examiner. Three randomly selected fields per slide were analyzed at 40x and 20x magnification, ensuring that selected fields had well-preserved tissue architecture without artifacts. Inflammatory cell count and area fraction were quantified using ImageJ, a public-domain image analysis software developed by the National Institutes of Health (Fig. 2).

### Inflammatory cell count and image analysis

Inflammatory cells were identified and quantified using ImageJ. Initially, five representative inflammatory cells were selected using the polygon selection tool, and their average size was determined (Fig. 2B). To exclude non-inflammatory structures, such as spindle-shaped fibroblasts, a circularity threshold of 0.3–1.0 (where 1.0 represents perfect roundness) was applied (Fig. 2C). The particle size range was configured; particles outside that range were excluded to ensure accurate cell counting (Fig. 2D, E).

### Assessment of new bone formation using goldner’s trichrome stain

Histomorphometric analysis was conducted to evaluate osteogenesis by measuring the surface area of new bone trabeculae within the periapical defect. Goldner’s trichrome stain (StatLab Medical, McKinney, TX, USA) was used to distinguish newly mineralized bone from surrounding tissues. The wand tracing tool in ImageJ was employed to automate the selection of new bone and matrix. The total surface area of new bone was measured, and the percentage of new bone formation was calculated relative to the total image area. Data were exported to an Excel sheet for statistical analysis.

### Immunohistochemistry for osteopontin detection

Osteopontin (OPN) is a sialoprotein that plays a crucial role in bone mineralization and remodeling. It is synthesized and secreted by preosteoblasts, osteoblasts, and osteocytes and is a key component of the mineralized extracellular matrix [14]. For OPN detection, 3 μm histological sections were incubated with a primary anti-osteopontin antibody (Abcam, ab8448, Cambridge, MA, USA) at a dilution range of 1:50–1:1,000. The primary antibody, a rabbit polyclonal antibody, exhibits species reactivity for mice, rats, and dogs. Sections were incubated overnight at 4 °C, followed by incubation with a biotin-conjugated bridging antibody (1:150 in blocking solution) for 30 min at 37 °C. Avidin-biotin complex (ABC) immunodetection was performed using a universal streptavidin-biotin peroxidase kit (Diagnostic BioSystem, Fremont, CA, USA), following the manufacturer’s instructions.

Positive immunolabeling was identified by brown-stained areas indicating OPN expression. For quantitative analysis, three randomly selected representative fields per root were examined at ×40 magnification. The wand tracing tool in ImageJ was used to automate the selection of positively stained cells and collagen-osteopontin matrix. The surface area of OPN expression was measured, and the percentage of positively stained tissue relative to the total area was calculated. Data were exported to Microsoft Excel for statistical analysis.

### Statistical analysis

Data distribution was assessed using the Kolmogorov-Smirnov and Shapiro-Wilk tests. Since the data were not normally distributed, non-parametric statistical methods were employed. Comparisons among the three experimental groups were performed using the Kruskal-Wallis test, followed by Dunn’s post hoc test with Bonferroni correction for multiple comparisons. To evaluate differences between the two observation time points (one month and four months), the Mann-Whitney U test was used for independent comparisons within each group. Data were presented as mean and median values. A *p*-value of < 0.05 was considered statistically significant. All statistical analyses were performed using SPSS^®^ Statistics Version 20 for Windows (IBM Corp., Armonk, NY, USA).

## Results

### Inflammatory cell count using hematoxylin and eosin stain

Independent of the observation period, the TotalFill BC Sealer group exhibited the significantly lowest inflammatory response (331.82 ± 147.47 cells), followed by the MTA Fillapex group (521.15 ± 185.28 cells), while the positive control group demonstrated the highest inflammatory cell count (*p* < 0.05) (Table 2). A significant reduction in inflammatory cell count was observed over time in all groups (*p* < 0.05).

**Table 2:** Inflammatory cell counts in the three groups at two-time intervals. Given are the means with standard deviations

Time intervals	TotalFill BC	MTA Fillapex	Control	*P*-value
**1 month**	473.08^Ca^ ± 44	702.35^Ba^ ± 37.3	767.96^Aa^ ± 55	0.001*
**4 months**	190.56^Cb^ ± 35	339.94^Bb^ ± 30.4	405.83^Ab^ ± 51	0.001*
*P*-value	0.001*	0.001*	0.001*	
Overall **Mean ± SD**	331.82^C^ ± 147.47	521.15^B^ ± 185.28	586.90^A^ ± 190.45	0.001*
overall **Median**	311 (102–538)	496 (258–786)	562 (319–849)	

Values with different capital superscript letters in the same raw are of statistically significant difference. Values with different small letters in the same column are of statistically significant difference. (*p* < 0.05)

**Fig. 3:**
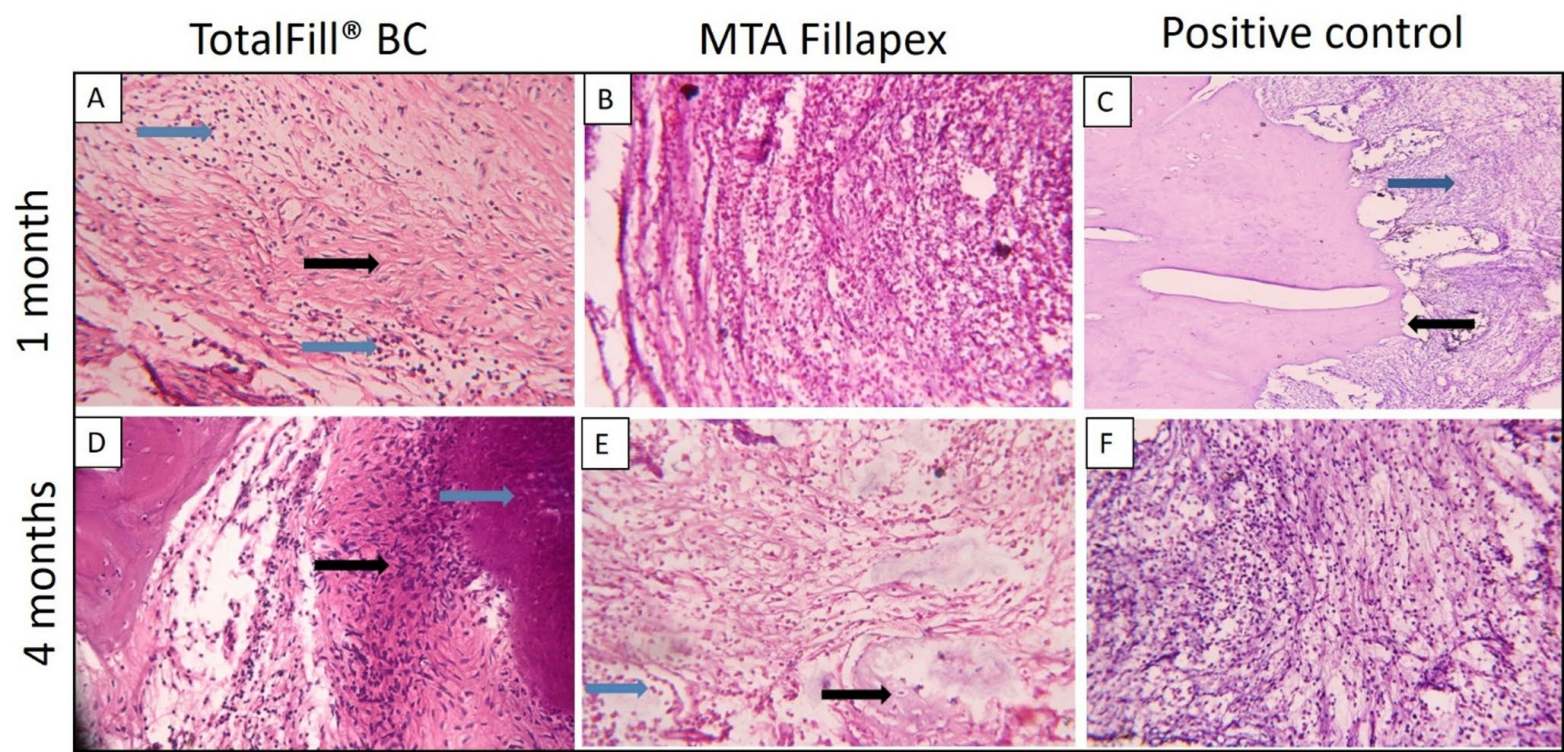
Photomicrographs of histological sections stained with H&E that show each experimental group and subgroup. (**A**) One-month TotalFill BC group, displaying inflammatory cells (blue arrow) and fibroblasts (black arrow) (x40). (**B**) At one month, the MTA Fillapex group showed a large number of inflammatory cells (x40). (**C**) At one month, the positive control group showed active resorption (black arrow) and a significant inflammatory reaction (blue arrow) close to the root apex. (**D**) TotalFill BC group at 4 months, displaying spindle-shaped undifferentiated mesenchymal cells in freshly generated tissue (black arrow) at the root apex (blue arrow) (x40). (**E**) Osteocytes embedded in newly formed bone (black arrow) and surrounded by chronic inflammatory cells (blue arrow) in the MTA Fillapex group at 4 months (x40). (**F**) Positive control group at 4 months, demonstrating persistent chronic inflammation (x40)

**Fig. 4:**
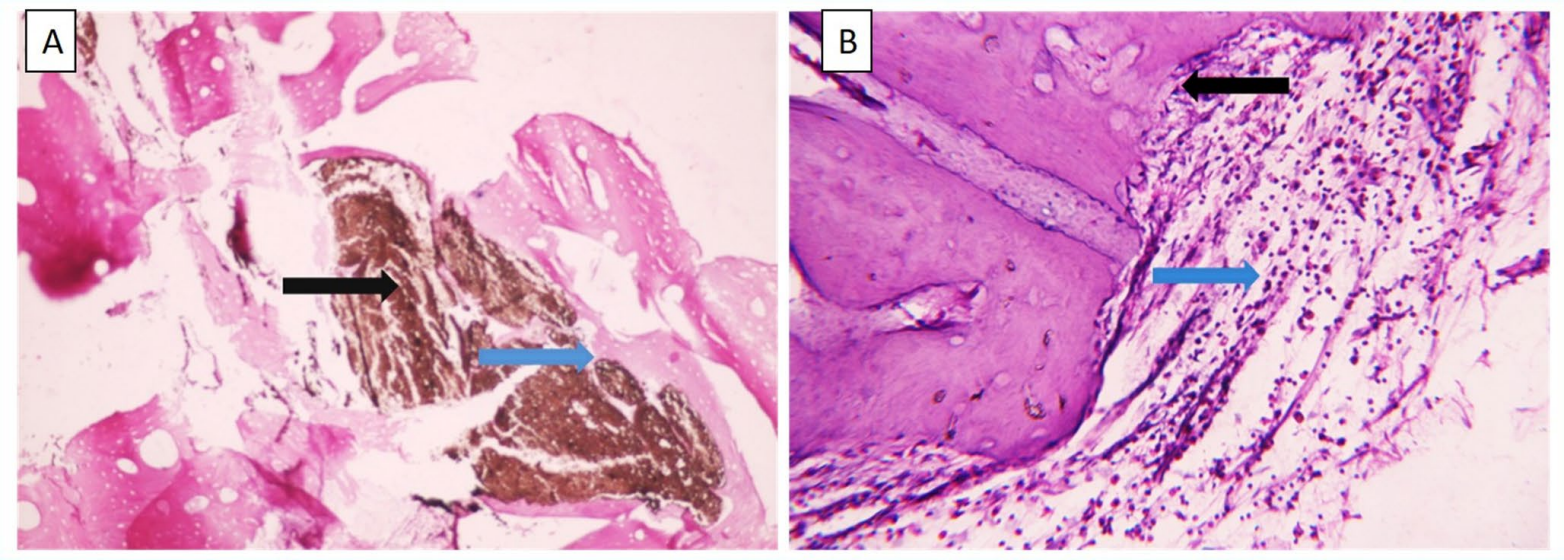
Photomicrographs of H&E-stained sections at 4 months (x20). (**A**) TotalFill BC group, showing traces of sealer material (black arrow) with no signs of inflammatory tissue reaction (blue arrow). (**B**) MTA Fillapex group, displaying a perforated apex with areas of resorption and remodeling (black arrow) and chronic inflammatory cells (blue arrow)

After one month, histopathological examination of the TotalFill BC Sealer group revealed a low inflammatory cell count, with spindle-shaped fibroblasts embedded within collagen fibers dispersed throughout the field (Fig. 3A). In contrast, the MTA Fillapex group exhibited a strong inflammatory response with severe inflammatory cell infiltration and active resorption of bone and cementum (Fig. 3B), while the positive control group showed extensive inflammatory cell infiltration and marked resorption of bone and cementum (Fig. 3C). At four months, the TotalFill BC Sealer group demonstrated minimal inflammatory cells and newly formed bone islands containing multiple osteocytes and undifferentiated mesenchymal cells within the connective tissue (Fig. 3D), with sealer remnants eliciting no inflammatory response (Fig. 4A). In the MTA Fillapex group, inflammation was reduced, but multinucleated osteoclasts and chronic inflammatory cells persisted at the bone borders, indicating an ongoing remodeling process (Figs. 3E and 4B). The positive control group continued to exhibit multinucleated osteoclasts and chronic inflammatory cells along the bone borders, consistent with a prolonged inflammatory state (Fig. 3F).

### New bone formation (Goldner’s trichrome stain)

**Fig. 5:**
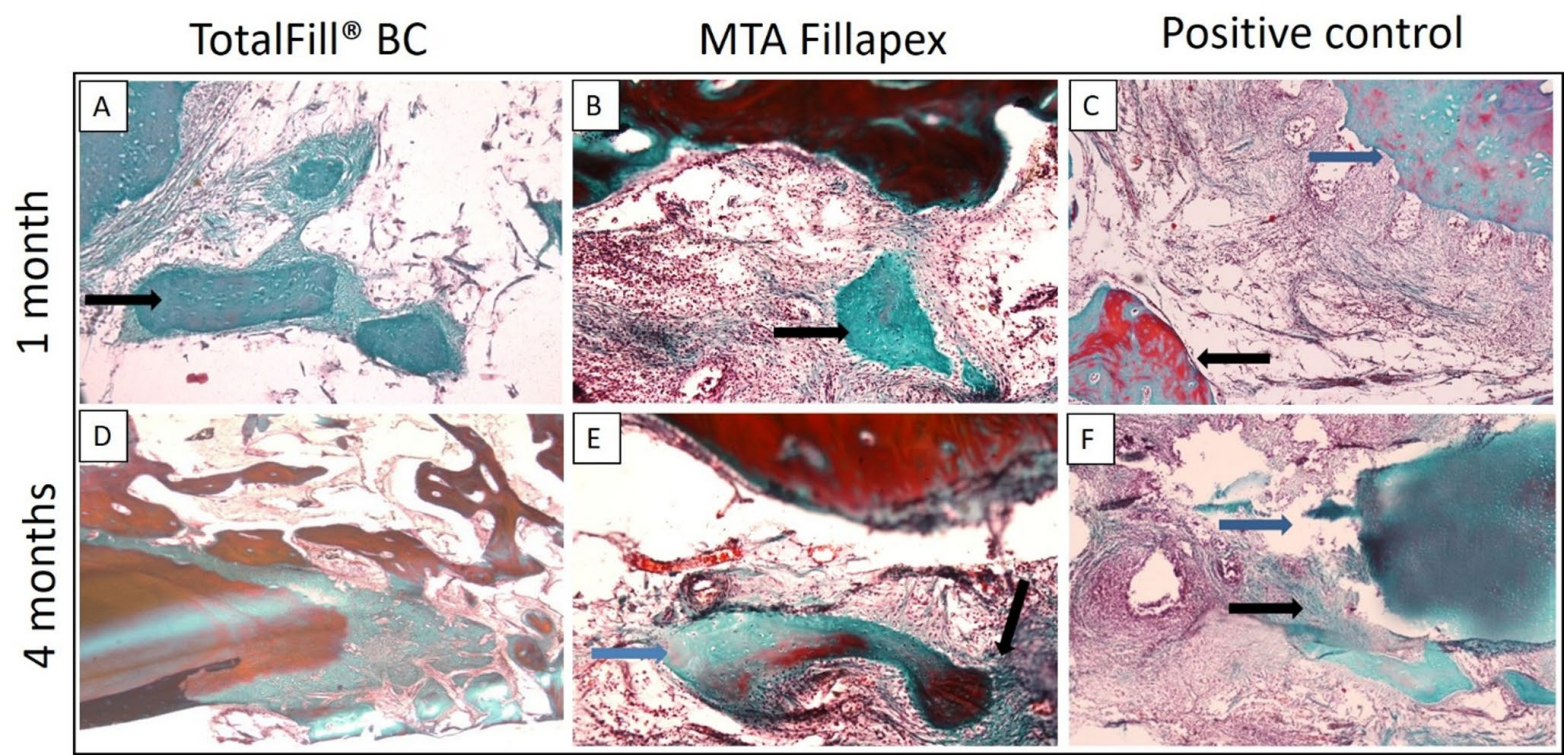
Photomicrographs of Goldner’s trichrome-stained sections at one and four months. (**A**) TotalFill BC, 1 month, showing islands of newly formed bone in green (black arrow) (x40). (**B**) MTA Fillapex, 1 month, showing newly formed bone (black arrow) (x40). (**C**) Positive control, 1 month, displaying the root apex (blue arrow) and remodeled old bone trabeculae (black arrow) (x40). (**D**) TotalFill BC, 4 months, showing newly formed mineralized tissue in close contact with the apex, surrounded by old bone (x20). (**E**) MTA Fillapex, 4 months, showing newly formed bone (blue arrow) surrounded by mineralized collagen matrix (black arrow) (x40). (**F**) Positive control, 4 months, showing the apical defect (blue arrow) surrounded by newly mineralized tissue (black arrow) (x20)

Overall, the TotalFill BC Sealer group exhibited the significantly highest percentage of new bone formation (33.98% ± 10.89) (*p* < 0.05). Additionally, a significant increase in new bone formation was observed over time in all groups (*p* < 0.05) (Table 3). After one month, histopathological examination of the TotalFill BC Sealer group showed apical defects surrounded by granulation tissue and foci of osteoid (Fig. 5A), while the MTA Fillapex group displayed areas of newly formed bone rich in osteocytes (Fig. 5B). At four months, the TotalFill BC Sealer group demonstrated extensive mineralized tissue in close contact with the root apex (Fig. 5D), whereas the MTA Fillapex group exhibited foci of osteoid deposition and a linear arrangement of newly mineralized collagen fibrils (Fig. 5E). In contrast, the positive control group showed minimal scattered osteoid tissue after one month, with traces of new bone formation and remodeled bone trabeculae observed after four months (Fig. 5C, F).

**Table 3:** Percentages of new bone formation in the three groups at two time intervals. Given are the means with standard deviations

Time intervals	TotalFill BC	MTA Fillapex	Control	*P*-value
**1 month**	23.43 ^Ab^± 1.56	13.24 ^Bb^± 1.78	9.21^Cb^ ± 1.31	0.001*
**4 months**	44.52 ^Aa^ ± 3.16	27.19 ^Ba^± 1.89	16.89^Ca^ ± 1.86	0.001*
**P-value**	0.001*	0.001*	0.001*	
**Overall Mean ± SD**	33.98^A^ ± 10.89	20.22^B^ ± 7.24	13.05^C^ ± 4.19	0.001*
**Overall Median**	33.98^A^ ± 10.89	20.22^B^ ± 7.24	13.05^C^ ± 4.19	

Mean values with different capital superscript letters in the same raw are statistically significantly different. Mean values with different small letters in the same column are statistically significantly different. (*p* < 0.05)

### Immunohistochemical results (osteopontin expression)

**Fig. 6:**
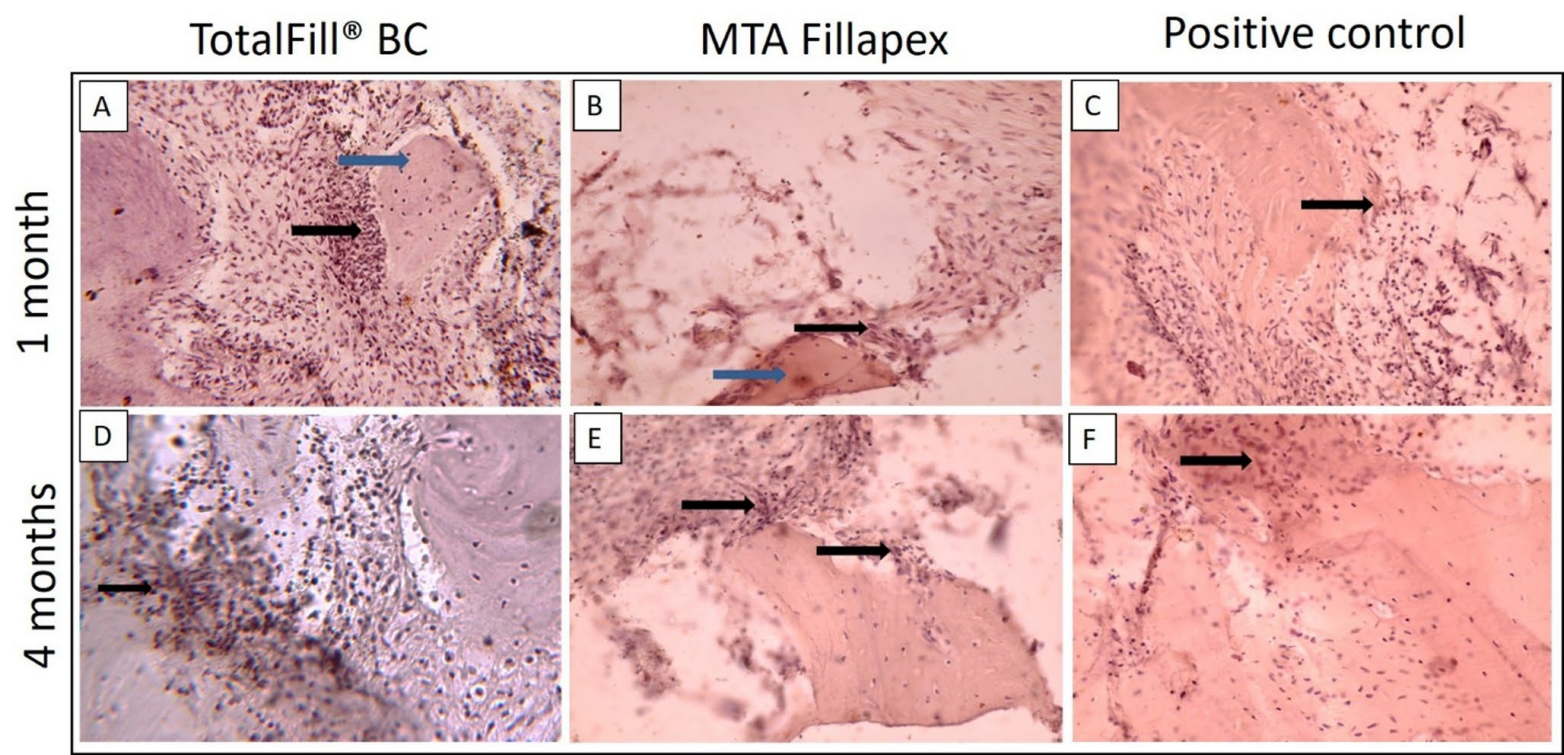
Photomicrographs of sections stained with anti-osteopontin at one and four months. (**A**) TotalFill BC, 1 month, displaying localized regions surrounding the newly produced bone (blue arrow) with extracellular osteopontin expression (black arrow) (x40). (**B**) MTA Fillapex, 1 month, displaying the freshly produced bone (blue arrow) surrounded by brownish-stained osteopontin (black arrow) (x40). (**C**) One-month positive control showing low expression of extracellular osteopontin (black arrows) (x40). (**D**) TotalFill BC at 4 months, showing intense osteopontin expression (black arrow) (x40). (**E**) MTA Fillapex, 4 months, showing mild extracellular osteopontin expression (black arrows) (x40). (**F**) Positive control, 4 months, showing moderate extracellular osteopontin expression (black arrows) (x40)

Regardless of any variable, the TotalFill BC Sealer group exhibited the significantly highest percentage of the surface area covered by anti-osteopontin staining (15.86% ± 4.64), followed by MTA Fillapex (10.40% ± 4.50) and the positive control group (5.57% ± 1.83) (*p* < 0.05). Additionally, significant differences were observed between the two-time points within each group (*p* < 0.05) (Table 4). After one month, the TotalFill BC Sealer group showed strong anti-osteopontin staining around newly deposited woven bone (Fig. 6A), whereas the MTA Fillapex group exhibited mild anti-osteopontin staining around newly formed bone (Fig. 6B). At four months, positive immunoreactivity for osteopontin was evident throughout the new bone borders in the TotalFill BC Sealer group (Fig. 6D), while the MTA Fillapex group displayed moderate osteopontin expression localized to areas adjacent to bone and cementum surfaces (Fig. 6E). In the positive control group, low immunoreactivity was observed at the bone trabeculae after one month, with moderate extracellular osteopontin expression beginning to appear after four months (Fig. 6C, F).

**Table 4:** Percentages of osteopontin surface area in the three groups at two time points. Given are the means with standard deviations

Time intervals	TotalFill BC	MTA Fillapex	Control	*P*-value
1 month	11.94^Ab^ ± 1.7	6.23^Bb^ ± 1.3	4.25^Cb^ ± 1.2	0.001*
4 months	19.78^Aa^ ± 2.9	14.56^Ba^ ± 1.9	6.90 ^Ca^ ± 1.3	0.001*
**P-value**	0.001*	0.001*	0.001*	
Overall Mean ± SD	15.86 A ± 4.64	10.40B ± 4.50	5.57 C ± 1.83	0.001*
overall Median	15.0 (9.4–24.6)	9.6 (2.2–18.7)	5.5 (2.4–8.5)	

Mean values with different capital superscript letters in the same raw are statistically significantly different. Mean values with different small letters in the same column are statistically significantly different. (*p* < 0.05)

## Discussion

The introduction of calcium silicate-based sealers was driven by the need for sealers with superior biocompatibility, the ability to promote healing, and optimal physicochemical properties. Calcium silicate-based sealers exhibit high hydrophilicity, allowing them to utilize physiological moisture within the canal and dentinal tubules to facilitate setting [15]. A notable characteristic of calcium silicate-based sealers is their delayed setting, which allows for extended antimicrobial activity. In their unset state, these sealers maintain a pH above 12, exerting antibacterial effects comparable to calcium hydroxide [16].

The present study aimed to evaluate, in vivo, the inflammatory response and osteoconductivity of a resin-free calcium silicate-based sealer (TotalFill BC) in comparison to a resin-based sealer (MTA Fillapex). A canine model was used, as dogs are considered an excellent experimental model for endodontic research [17]. Their dentition allows for the simulation of masticatory forces and thermal cycling variations, closely resembling clinical conditions. Additionally, canine tissue responses have been shown to be similar to those of humans, further supporting their suitability for this study [18].

The mandibular premolars of dogs were selected for this study since they are not in direct contact with maxillary teeth, minimizing the influence of occlusal stress on any apical periodontitis that may develop post-procedure. Additionally, the root canals of the second, third, and fourth premolars are sufficiently wide to allow proper instrumentation [19]. However, unlike the human apical anatomy, canine teeth feature an apical delta with ramifications. This may influence periradicular tissue responses. To standardize conditions and facilitate periapical evaluation, the apical cementum layer was mechanically perforated at the anatomic apex [20].

In the present study, a semi-automated digital analysis was employed to minimize bias, inconsistency, and human errors associated with visual assessment and eye strain. While scoring systems are widely used, they remain inherently subjective as they rely on the assessors’ interpretations. In contrast, digital analysis offers a more objective approach, particularly when evaluating large datasets [21]. This method enables precise quantification of multiple parameters, such as cell density, average cell size, and area fraction [22]. However, both methods have limitations, including potential bias during image capture and variability in threshold adjustments during digital analysis, which should be carefully considered.

The null hypothesis of the present study was rejected, as overall the image analysis of hematoxylin and eosin (H&E)-stained sections revealed that the resin-free TotalFill BC Sealer exhibited the significantly lowest inflammatory response and inflammatory cell count at the two-time points compared to MTA Fillapex and the control group. This outcome may be attributed to the sealer’s high pH and effective sealing ability. These findings regarding inflammatory cell count align with those of a previous in vivo study [23] and a study conducted on a 3D-culture model of human osteoblast-like cells [24]. In the present study, healing with bone or cementum-like tissue on the peripheral root-end surface was observed in some samples filled with TotalFill BC, consistent with findings from a previous study [25]. Additionally, the significant reduction in inflammation over time, particularly in response to the resin-free sealer, aligns with the findings of Bueno et al. [26], further supporting the biocompatibility and favorable tissue response of resin-free calcium silicate-based materials. Furthermore, compared to a resin-based sealer, TotalFill BC has shown anti-inflammatory properties and a markedly reduced generation of IL6 and IL8 in a recent study [27].

In this study, the resin-based MTA Fillapex sealer elicited a high inflammatory response, particularly during the one-month evaluation period. In some samples, a fibrous capsule was observed surrounding the extruded MTA Fillapex sealer. However, after four months, the inflammatory reaction had significantly diminished. These findings are consistent with a previous study by Assmann et al. [28], in which MTA Fillapex was injected directly into a bony cavity in rats to simulate sealer extrusion into the periapical region. The study reported significantly higher neutrophil counts at seven days compared to 90 days. Similarly, another study [29] demonstrated that MTA Fillapex induced a persistent and severe inflammatory response lasting up to 90 days compared to a resin-free calcium silicate sealer (iRoot SP; Innovative BioCreamix, Vancouver, Canada). Additionally, an in vivo study using a canine model [17] reported that MTA Fillapex caused moderate to severe inflammatory reactions in the periapical region of dogs’ premolars even after six months.

The pro-inflammatory reactions observed in these studies may be attributed to the chemical composition of MTA Fillapex, which is primarily based on salicylate resin. The presence of resin has been shown to reduce cell survival rates and significantly increase cytotoxicity, showing a threefold increase in cytotoxicity compared to resin-free calcium silicate sealer [30]. In contrast, a previous study conducted on rats [31] reported only a mild inflammatory reaction at 30 and 90 days. This discrepancy may be due to the use of a subjective scoring system for histological evaluation, as well as differences in interpretation, where the inflammatory response was considered mild compared to earlier time points, such as 7 and 15 days.

Several studies [32–34] have investigated the osteogenic and mineralization potential of calcium silicate-based sealers, the majority of which were conducted in vitro. In the present in vivo study, osteoconductivity was assessed using Goldner’s trichrome stain, which differentiates tissue components based on color and morphology [35]. This staining technique allows clear discrimination between the new bone matrix (osteoid) and mature bone matrix [36], with newly mineralized tissue appearing as a compact green color. Even after long-term interventions, Goldner’s Trichrome stain enables the detection of newly formed bone [35]. Previous studies have shown that the first trabeculae of osteoid and new woven bone can be observed within two weeks, while significant bone formation typically begins after four weeks and can be assessed for up to six months [37].

In the present study, TotalFill BC demonstrated the highest percentage of osteoid tissue formation, with statistically significant differences observed at both one and four months compared to MTA Fillapex and the control groups. This enhanced osteoconductivity may be attributed to the material’s high biocompatibility and its ability to support osteoblastic cell attachment and proliferation [34, 38]. Similar findings were reported by Rifaey et al. [39], who found that bioceramic endodontic materials promoted osteoblastic differentiation in a 3D culture system, suggesting their potential role in enhancing bone formation. Furthermore, compared to cells grown in either unconditioned or osteogenic culture conditions, a recent study found that TotalFill BC markedly enhanced the development of calcified nodules on human periodontal ligament stem cells [40].

The superior osteogenic potential of calcium silicate-based materials may be attributed to their ability to stimulate angiogenesis and promote osteogenesis through the release of bioactive byproducts [32, 41].

Although new bone formation increased in MTA Fillapex samples after four months, it remained significantly lower than in the TotalFill BC group. These findings align with previous reports suggesting that the presence of MTA in MTA Fillapex does not enhance bone tissue repair [28]. However, contrary to these in vivo results, an ex vivo bone development model demonstrated enhanced osteogenic activity with two different MTA-based sealers [42]. This discrepancy may be attributed to differences in sealer composition, particularly the proportion of MTA relative to other additives, such as resin, which may influence their bioactivity and osteogenic potential.

The third histological assessment in the present study utilized immunohistochemistry, a technique for detecting specific antigens (e.g., intracellular or extracellular proteins) in tissue sections by exposing them to antibodies that selectively bind to the target antigens. Detection is facilitated by chromophores linked to these antibodies. In this study, osteopontin (OPN) was used as an antigen marker for osteoconductivity. OPN is a non-collagenous sialoprotein and a key component of the mineralized extracellular matrices of bones and teeth [43]. It is highly concentrated at cement lines, where preexisting and newly formed bone meet, as well as at bone-cell interfaces (laminae limitantes). OPN is expressed by osteocytes, osteoblasts, osteoclasts, and hypertrophic chondrocytes. Additionally, it is present in various non-mineralized tissues and cells, such as periodontal and gingival fibroblasts. OPN is assumed to be a multifunctional protein that is involved in osteoblastic proliferation, bone formation, and bone turnover. It can regulate initial events during bone remodeling, such as bone cell adhesion, osteoclast function, and matrix mineralization [44].

In this study, immunohistochemical staining was performed based on previous evidence suggesting that OPN plays a role in the initial formation and/or calcification of the bone matrix [45]. OPN expression was measured in the periapical area within selected representative fields, as well as at the marginal regions of newly formed osteoid and woven bone matrix, and along the reversal lines, following previously established methods [37]. The exact origin of OPN could not be definitively determined, as it may be released by a variety of cells, including osteoclasts [37]. Therefore, any areas exhibiting signs of active bone resorption were excluded from the analysis in this study. Active resorption sites were identified by the presence of osteoclasts. In this study, OPN was detectable for up to four months. Previous evidence supports that OPN is strongly expressed near the newly formed bone, even up to 5–6 months post-treatment [37], further suggesting that this protein plays a significant role in bone remodeling.

TotalFill BC induced significantly greater expression of OPN around the bone trabeculae after one and four months compared to both the MTA Fillapex and control groups. OPN was observed in the connective tissue adjacent to newly formed hard tissue, as well as in the cells and matrix of the newly formed tissue. This supports the potential role of OPN in osteogenesis. A previous study demonstrated that the healing effect of sodium hyaluronate in sockets was, in part, attributed to the stimulation of OPN expression [14]. In the current study, the MTA Fillapex group showed low expression of OPN after the first month, with a significant increase observed after four months. The control group exhibited the lowest expression of OPN at both time points. These findings are supported by a previous study [46] that examined the ability of calcium silicate-based sealers implanted in rats’ subcutaneous tissue to express OPN.

On the whole, the results of the present study corroborate the findings of a systematic review on pulp-capping materials [10] and a laboratory study assessing endodontic repair materials [11]. Both studies concluded that resin-free calcium silicate-based materials are more biocompatible, possess a greater potential to induce proliferation and adhesion of host stem cells, and show a better promotion of healing processes in surrounding tissues (either pulp or as in this study; periradicular tissue) than resin-based calcium silicate-based materials. A previous study showed that over 4 weeks, MTA Fillapex was significantly more cytotoxic than an epoxy resin-based sealer, and the authors assumed that this was due to some toxic components of MTA Fillapex, such as salicylate resin and diluting resin [47]. Similar results and explanations have been reported by other authors [48–50]. Thus, the findings of the present study are in good agreement with previously published data.

Besides the limitation mentioned above associated with the semi-automated digital analysis, this study may have other limitations, as no established sealer (e.g., an epoxy resin-based sealer as the accepted gold standard) was used for comparisons. The reasons for refraining from establishing a further control group are twofold. Firstly, a recent study already reported that epoxy resin-based sealers do not possess any osteogenic potential [51]. Secondly, the main research question of this study was to evaluate potential differences between resin-free and resin-based calcium silicate-based sealers. Therefore, in order to avoid unnecessary sacrifice of animals, no further sealer group was established. Moreover, the current experimental setup did not allow to exactly standardize the amount of extruded sealer, which might represent another limitation. However, inclusion criteria for further assessments of all teeth were that sealer extrusion was confirmed radiologically and that extrusion was at least located 1 mm beyond the apex. Thus, direct contact of the sealer with the periapical tissues was ensured for all cases.

## Conclusion

The resin-free TotalFill BC Sealer exhibited superior osteoconductivity and biocompatibility compared to the resin-based MTA Fillapex sealer at both one and four months. The inflammatory response to TotalFill BC was minimal and decreased over time, supporting its favorable tissue response. Limited sealer extrusion could accelerate these favorable outcomes. MTA Fillapex induced a higher inflammatory response and showed lower osteoconductivity, despite some improvement in bone formation over time. Further randomized clinical studies are needed to confirm these results in different clinical settings.

## Data Availability

Data is provided within the manuscript or supplementary information files.All data used and/or analyzed during this research are available from the corresponding author upon reasonable request.
